# Trends and health equity in environmental sustainability publications in major anaesthesia journals

**DOI:** 10.1111/anae.16467

**Published:** 2024-11-04

**Authors:** Marco S. Fabus, Søren Kudsk‐Iversen

**Affiliations:** ^1^ University of Oxford Oxford UK; ^2^ Oxford University Hospitals NHS Trust Oxford UK

Climate hazards are associated with health disparities, creating vicious cycles that disproportionately impact marginalised groups [1]. There is increasing interest in healthcare sustainability, including in anaesthesia, with the National Health Service (NHS) committed to ‘net zero’ carbon emissions by 2040. Peri‐operative healthcare sustainability interventions can broadly be divided into four categories [2]. First, and most impactful, is disease prevention. Second is patient empowerment interventions, including surgical prehabilitation. Third, moving healthcare towards lean‐care systems, avoiding wasteful practices. Finally, doctors can switch to low‐carbon alternatives, such as reusable instruments.

We describe the results of a rapid review, where we considered what types of publications related to environmental sustainability are being published in anaesthesia journals, and to what extent these sustainability publications consider health inequity. The full, pre‐registered methodology has been published previously [3].

Briefly, we conducted a literature search in PubMed on 31/01/2024, focusing on English language articles in anaesthesia journals listed in the InCites Journal Citation Reports (N = 65), using a broad query string with terms related to climate change; greenhouse gases; and sustainability (Mesh terms included). After screening, we extracted information about primary outcomes; categories of interventions mentioned (prevention, patient empowerment, lean‐care systems and low‐carbon alternatives); first‐author affiliated institution location and its World Bank income region; and text relating to inequality, inequity, or climate justice. We defined health inequality as a difference in measurable health outcomes between individuals or groups; health inequity as a specific type of health inequality that is preventable, unnecessary and unjust; and climate justice as the approach that recognises inequities and designs interventions to correct them. After extraction, we used custom Python 3.8 code to extract descriptive statistics and a word cloud generator to analyse climate justice text (available in online Supporting Information Figure S1).

We identified 199 publications on sustainability in 27/51 (53%) PubMed‐indexed journals (online Supporting Information Table S1). Most sustainability publications (175/199, 88%) were in ten journals, and 80/199 (40%) of publications presented original research. Most publications (177/199, 89%) focused on low‐carbon alternatives, 66/199 (33%) discussed ‘lean‐care systems’, 11/199 (6%) discussed patient empowerment; and 6/199 (3%) discussed disease prevention. Visualised within the ‘Pyramid of Impact’ (Fig. 1), the focus was mostly on in‐theatre mitigation. Patient empowerment interventions included broadening the anaesthetic consent process to include information and choices about environmental impact; more patient education and engagement; using patient‐reported outcomes; and including patient representatives in research and policy meetings. Disease prevention interventions included prehabilitation; broadening pre‐ and postoperative clinics towards preventative management; ‘getting it right first time’ principles (GIRFT); and supporting active transport in patients. No studies quantified the environmental impact of these interventions.

**Figure 1:**
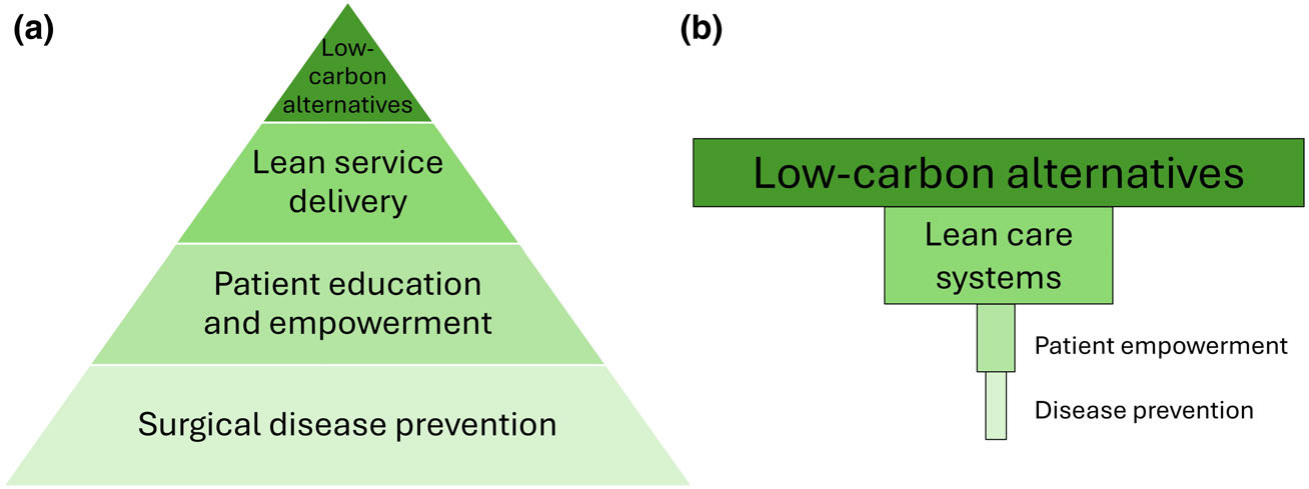
Stratification of interventions in sustainability publications. (a) The ‘pyramid of impact’ adapted from Rizan et al. [2]. (b) The actual distribution of papers, with the rectangle width of each category scaled to the proportion of papers discussing such interventions.

Most sustainability publications (194/199, 98%) had first authors affiliated with high‐income country institutions. Article type distribution varied between countries and journals, with *Anaesthesia* having the largest proportion of articles discussing patient empowerment (5/29, 17.2%). All articles discussing patient empowerment or disease prevention were from 2019 onwards. Few publications (32/199, 16%) had text relating to inequality or inequity, and none met our definition as talking about climate justice. Such discussions focussed predominantly on inequality, and mostly on between‐country differences. Inequality was mentioned largely to indicate relevance of studying sustainability or as a limitation of generalisability. No original research related study findings to their impact on inequality or inequity.

Our work highlights a disproportionate focus on in‐theatre mitigation. Some of this work has been highly impactful, e.g. the UK Nitrous Oxide Project. However, prevention and empowerment have been suggested as the most impactful categories of reducing peri‐operative environmental impact [4, 5]; we need more original research to substantiate this [6, 7]. Several interventions are ripe for testing, including prehabilitation; broadening shared decision making to include environmental information; and redefining peri‐operative care to encompass prevention. Limitations of our work include only using one database and publications in English.

Anaesthetists are often already working on these interventions. Crucially, however, they are not being recognised or tested for their environmental benefits. In our view, lack of equity consideration is also a missed opportunity. Researchers looking to conduct clinical interventional studies in sustainability should proactively centre equity, e.g. by using PROGRESS‐Plus [8]. Climate change worsens health inequities; considering them in isolation risks failing to address either effectively.

## Supporting information


**Figure S1.** Word frequency cloud of text related to climate justice or social inequality in sustainability publications within anaesthesia journals from 32 papers that discussed the topic.

**Table S1.** Extended results showing article type breakdown by country.
