# Supplementary material for: Trends and health equity in environmental sustainability publications in major anaesthesia journals
Source: Anaesthesia. 2024 Nov 4;80(1):115–6. doi: 10.1111/anae.16467 (PMC11617129; doi:10.1111/anae.16467)

**Figure S1:** Word frequency cloud of text related to climate justice or social inequality in sustainability publications within anaesthesia journals, from n=32 papers that discussed the topic.


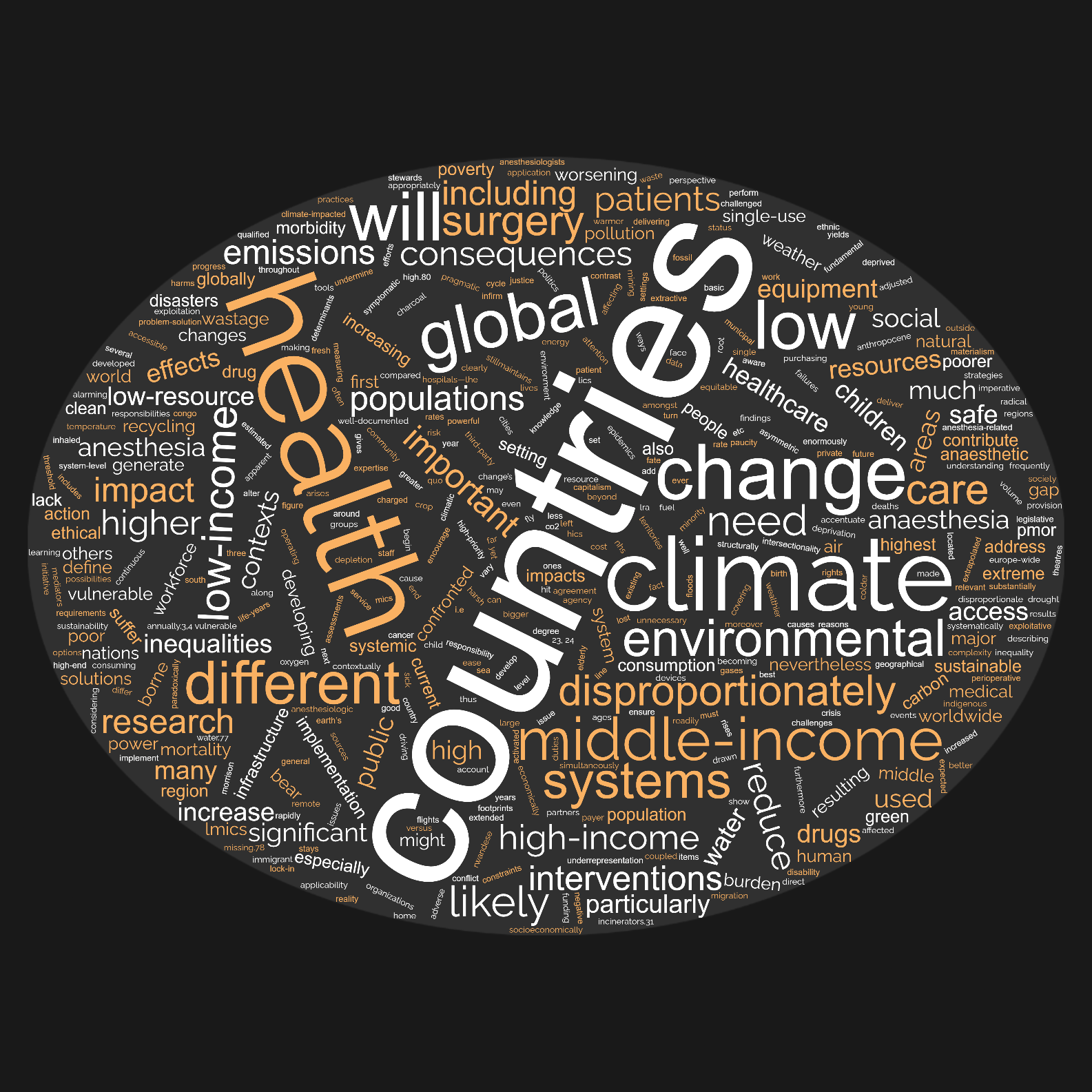

Supplement: Supplementary file 1 — Figure S1. Word frequency cloud of text related to climate justice or social inequality in sustainability publications within anaesthesia journals from 32 papers that discussed the topic. [file ANAE-80-115-s002.docx]
