# Supplementary material for: Trends and health equity in environmental sustainability publications in major anaesthesia journals
Source: Anaesthesia. 2024 Nov 4;80(1):115–6. doi: 10.1111/anae.16467 (PMC11617129; doi:10.1111/anae.16467)
Supplement: Supplementary file 2 — Table S1. Extended results showing article type breakdown by country. [file ANAE-80-115-s001.docx]

**Table S1:** Extended results showing article type breakdown by country

| Country | Total | Original research | Review | Correspondence | Editorial |
| --- | --- | --- | --- | --- | --- |
| USA | 48 | 37.50% | 12.50% | 37.50% | 18.80% |
| UK | 44 | 27.30% | 20.50% | 43.20% | 27.30% |
| Australia | 31 | 48.40% | 9.70% | 38.70% | 9.70% |
| Canada | 17 | 41.20% | 11.80% | 52.90% | 5.90% |
| France | 17 | 58.80% | 11.80% | 41.20% | 17.60% |
| Germany | 7 | 57.10% | 42.90% | 14.30% | 0.00% |
| Switzerland | 4 | 75.00% | 25.00% | 0.00% | 0.00% |
| Singapore | 4 | 75.00% | 25.00% | 50.00% | 0.00% |
| Belgium | 4 | 25.00% | 25.00% | 50.00% | 0.00% |
| Spain | 3 | 0.00% | 33.30% | 33.30% | 0.00% |
| Italy | 3 | 66.70% | 0.00% | 33.30% | 33.30% |
| Netherlands | 3 | 0.00% | 33.30% | 0.00% | 33.30% |

| **Denmark** | 2 | 50.00% | 50.00% | 0.00% | 0.00% |
| --- | --- | --- | --- | --- | --- |
| **NZ** | 2 | 100% | 0.00% | 50.00% | 0.00% |
| **India** | 2 | 50.00% | 50.00% | 0.00% | 0.00% |
| **Norway** | 1 | 0.00% | 0.00% | 0.00% | 100% |
| **Portugal** | 1 | 0.00% | 0.00% | 100% | 0.00% |
| **Japan** | 1 | 0.00% | 0.00% | 100% | 0.00% |
| **Iran** | 1 | 0.00% | 0.00% | 100% | 0.00% |
| **Sweden** | 1 | 0.00% | 0.00% | 0.00% | 100% |
| **Turkey** | 1 | 100% | 0.00% | 0.00% | 0.00% |
| **Brazil** | 1 | 0.00% | 100% | 0.00% | 0.00% |
| **Monaco** | 1 | 0.00% | 0.00% | 0.00% | 100% |

| TOTAL | 199 | 40% | 17% | 38% | 17% |
| --- | --- | --- | --- | --- | --- |
